# Kyste mucoïde du ligament croisé antérieur: à propos d’un cas

**DOI:** 10.11604/pamj.2016.24.331.10196

**Published:** 2016-08-30

**Authors:** Louaste Jamal, Taoufik Cherrad, Hicham Bousbaa, Hassan Zejjari, Mohammed Ouahidi, Larbi Amhajji, Khalid Rachid

**Affiliations:** 1 Service de Chirurgie Orthopédique et Traumatologique, Hôpital Militaire Moulay Ismail, BP 50000 Meknès, Maroc

**Keywords:** Kyste mucoïde, ligament croisé antérieur, arthroscopie, Mucoid cyst, anterior cruciate ligament, arthroscopy

## Abstract

Les kystes mucoïdes du ligament croisé antérieur (LCA) sont rares. Ils sont souvent asymptomatiques et méconnus. Leur expression clinique est polymorphe et c'est l'imagerie par résonnance magnétique qui permet de poser le diagnostic qui est confirmé par l'histologie. La résection arthroscopique constitue le traitement de référence. On rapporte un cas de kyste mucoïde du LCA traité par résection arthroscopique.

## Abstract (en)

Anterior cruciate ligament (ACL) mucoid cysts are rare. They are often asymptomatic and unrecognized. Their clinical expression is polymorphic and MRI allows for an early diagnosis confirmed by histology. Arthroscopic resection is the gold standard treatment. Our study reports a case of ACL mucoid cyst treated with arthroscopic resection.

## Introduction

Les kystes mucoïdes du genou sont des formations kystiques qui peuvent être intra-articulaire (ligaments croisés, graisse infrapatellaire de Hoffa) ou para-articulaire (dans l'os, dans le tissu cellulo-graisseux juxta-articulaire, dans les muscles ou en sous-périosté). Le kyste intra-articulaire est rare, sa prévalence à l'IRM est de 1.3% et de 0.6% à l'arthroscopie [1]. Il touche préférentiellement le ligament croisé antérieur (LCA), souvent méconnue car il n'est pas toujours symptomatique. Le tableau clinique habituel du kyste du LCA comprend des gonalgies et une limitation des mouvements articulaire. L'IRM est l'examen de choix, il permet de distinguer les lésions infiltrantes, autrement appelées hypertrophie, ou dégénérescence mucoïde, ou encore pseudokyste infiltrant du LCA, des lésions purement kystiques [2]. Le traitement peut être arthoscopique ou par ponction infiltration scano- ou échoguidée [3]. Nous rapportons un cas de kyste mucoïde du LCA traité chirurgicalement par une résection arthroscopique.

## Patient et observation

**Figure 1:**
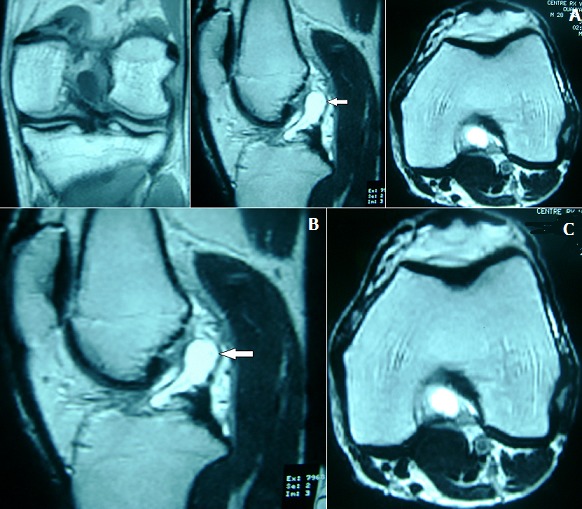
A) IRM du genou montrant une formation kystique au dépend du LCA en hyposignal T1 sur la coupe coronale; B) IRM du genou en franc hypersignal T2 sur la coupe sagittale; C) IRM du genou avec comblement de l’échancrure intercondylienne sur la coupe axiale

**Figure 2:**
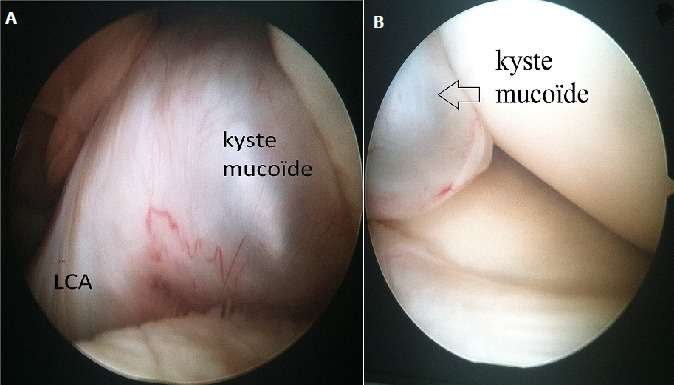
(A,B) vue arthroscopique montrant un kyste mucoïde du LCA

**Figure 3:**
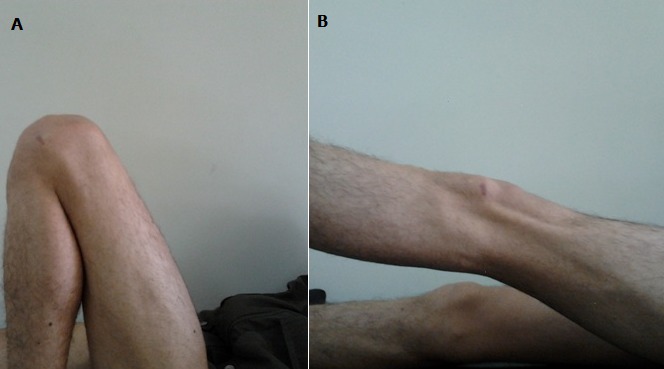
A) résultats fonctionnel postopératoire; B) flexion; C) extension

Notre patient est un homme âgé de 38 ans, ayant comme antécédent un traumatisme du genou gauche survenu au cours d'un match de football il y a 6 mois, avec apparition de gonalgie chronique de type mécanique, d'aggravation progressive et une limitation de la flexion et de l'extension du genou gauche. L'examen clinique trouve un flessum du genou gauche avec une mobilité articulaire de 10°/100°, et une douleur à la palpation de la face postérieure du genou gauche. Par ailleurs on ne note pas de choc rotulien, les signes méniscaux sont négatifs et le testing ligamentaires est normal. La radiographie standard du genou est sans particularité sur les incidences face et profil, tandis que l'IRM montre sur l'acquisition en plan coronal en pondération T1 et en plan sagittal et axial en séquence pondérée T2 DP la présence d'une formation grossièrement ovalaire, mesurant 33x14 mm, bien limitée, à contours réguliers, en hyposignal T1 et hypersignal franc T2 (liquidien) homogène, au dépend du LCA dont les fibres paraissent continues et gardent une orientation normale ce qui a évoquer le diagnostic d'un kyste du LCA Figure 1. L'exploration arthroscopique a permis d'objectiver et de réséquer le kyste mucoïde au niveau du LCA Figure 2, par ailleurs le LCA était bien tendu et continu à l'examen par le palpateur. Les ménisques étaient intacts. Le diagnostic de kyste mucoïde a été confirmé par l'examen anatomopathologique. L'évolution a été marquée par la disparition de la douleur au bout de 15 jours et la récupération des amplitudes articulaires **Figure 3**.

## Discussion

Les kystes mucoïdes du genou sont des formations kystiques néoformées constituées d'une ou plusieurs cavités contenants du liquide mucoïde très visqueux et entourées d'une paroi fibroconjonctive. Les kystes touchants les ligaments croisés sont rares. Leur étiologie n'est pas encore bien élucidée, mais leur localisation souvent dans des zones sous contrainte mécanique continue et la fréquence des antécédents de genou traumatique font évoquer une origine traumatique forte semblable [4]. Ces kystes sont fréquemment silencieux. Quand ils deviennent symptomatique ; ils entrainent des douleurs progressive, s'aggravant avec le temps et souvent postérieur dans le creux poplité. Ces gonalgies profondes sont fréquemment associées à une limitation de la flexion et/ou de l'extension avec parfois des épisodes de blocage voire d'hémarthrose. La douleur est généralement secondaire à l'effet de masse du kyste du LCA dans l'échancrure postérieure ou à un impignement entre le LCA et le LCP [4, 5]. L'IRM est l'examen de choix pour le diagnostic positif et des lésions associées. Les kystes du LCA sont fusiformes, orientés dans le grand axe du ligament, de signal liquidien avec parfois une expansion kystique multiloculaire. Lorsque le kyste est de situation intraligamentaire pure, le ligament apparaît élargi et en éventail avec signe de la «branche de céleri » [6]. Une aspiration scanno ou écho guidée peut être proposée comme moyen thérapeutique mais elle n'est pas accessible aux localisations profondes et expose au risque de récidive [7]. Ainsi la résection arthroscopique reste la méthode de choix, vu son caractère mini-invasive, ses bons et excellents résultats sur la douleur et la mobilité articulaire et de même que sur le taux de récidive qui est nul pour la plupart des auteurs. Elle permet également de traiter les lésions associées.

## Conclusion

Le diagnostic clinique de ces kystes est difficile en raison de leur symptomatologie polymorphe, par contre l'IRM permet le diagnostic positif grâce à sa sensibilité et sa spécificité. La résection arthroscopique constitue le traitement de choix vu ses bons résultats fonctionnels et un taux de récidive quasi nulle.
